# On the methodological unification in electroencephalography

**DOI:** 10.1186/1475-925X-4-15

**Published:** 2005-03-04

**Authors:** Piotr J Durka

**Affiliations:** 1 Institute of Experimental Physics, Warsaw University, ul. Hoża 69 00-681 Warszawa, Poland

## Abstract

**Background:**

This paper presents results of a pursuit of a repeatable and objective methodology of analysis of the electroencephalographic (EEG) time series.

**Methods:**

Adaptive time-frequency approximations of EEG are discussed in the light of the available experimental and theoretical evidence, and applicability in various experimental and clinical setups.

**Results:**

Four lemmas and three conjectures support the following conclusion.

**Conclusion:**

Adaptive time-frequency approximations of signals unify most of the univariate computational approaches to EEG analysis, and offer compatibility with its traditional (visual) analysis, used in clinical applications.

## 1 Background and main ideas

### 1.1 Historical background

"Animal electricity" has been subject to scientific research since the end of the 18th century, when Galvani and Volta performed their famous experiments [1]. Electrical activity of the brain was first mentioned in 1875, in a grant report by R. Caton [2]. In 1929 the first electroencephalogram (EEG) was recorded from the surface of human scalp by Hans Berger [3].

Year 1935 witnessed birth of the major fields of today's clinical electroencephalography. F. Gibbs and H. Davis [4] showed association of 3/sec spike-wave complexes in EEG with epileptic petit mal absences, and A. L. Loomis et al [5] methodically studied human sleep EEG patterns and the stages of sleep. Also in 1935, the first electroencephalograph (Grass Model I) started the era of contemporary EEG recording: galvanometers, used in earlier decades to record EEG traces on photographic paper, were replaced by 3-channels preamplifier, and the recording was drawn by ink writer on rolls of paper. These rolls were later replaced by folded paper, and, currently, by digital storage and display of EEG traces. Also, contemporary amplifiers provide higher sensitivity and number of channels, but all these changes are quantitative rather than qualitative.

Finally, by the end of forties, Dawson [6] recorded first evoked potentials. He constructed an advanced mechano-electrical (analog) device for averaging brains potentials triggered by a stimulus [7]. Averaging was indispensable to show the event-related activity, which is normally invisible in the on-going EEG background.

These techniques, combined with *visual *analysis of recorded EEG traces, constitute the canon of contemporary clinical electroencephalography.

### 1.2 Computational techniques of EEG analysis

First attempts were made by Hans Berger in 1932 [8]; he was assisted by the physicist Dietsch, who applied Fourier analysis to short EEG sections. Then, quoting [9]: *The 1950s saw the early generation of automatic frequency analyzers approaching and eventually saw the end of these magnificent but mostly unused machines*.

Digital storage of the EEG time series opened unlimited possibilities of offline analysis, and triggered significant efforts towards algorithmic solutions. Studies published in last decades cover the whole spectrum of possible signal processing methods. This is partially due to the fact, that "first" application of given signal processing method to an old experimental paradigm or dataset usually fulfills the requirement of scientific novelty, and justifies publication.

### 1.3 The need for a unification

As mentioned in section 1.1, nowadays technology of EEG recording is quite satisfactory: we can record simultaneously more channels than the number of electrodes which would fit on a scalp, with sampling (in both time and amplitude) exceeding the assumed information content of the signal. Unresolved issues relate to the analysis of these huge amounts of data.

One of the major problems in biomedical sciences is the inter-subject variability. Especially in neurosciences, it may dramatically exceed the effects attributed to the investigated phenomena. This naturally leads to an unrealistic postulate that, to draw significant conclusions, each new hypothesis or method must be evaluated on a sufficiently large dataset, that is involving enough (probably at least thousands) human subjects. Otherwise, a coherent progress may be achieved only via comparison of different studies, performed and analyzed in a compatible way.

The only paradigm, up to now applied to a significant amount of cases, is the classical, visual analysis of EEG, involved in clinical and research applications. *Classic EEG evaluation always involved measuring frequency and/or amplitude with the help of simple rulers *[10]. Some of the EEG structures are even defined using terms like "cycles per second" and "microvolts" [11]. Nevertheless, new methods proposed for EEG analysis are mostly incompatible with such description. Therefore, they cannot be directly related to this indispensable knowledge base. Also, results offered by most of the advanced methods are seldom compatible with each other, so they do not seem to form a new, coherent knowledge base.

**Figure 1:**
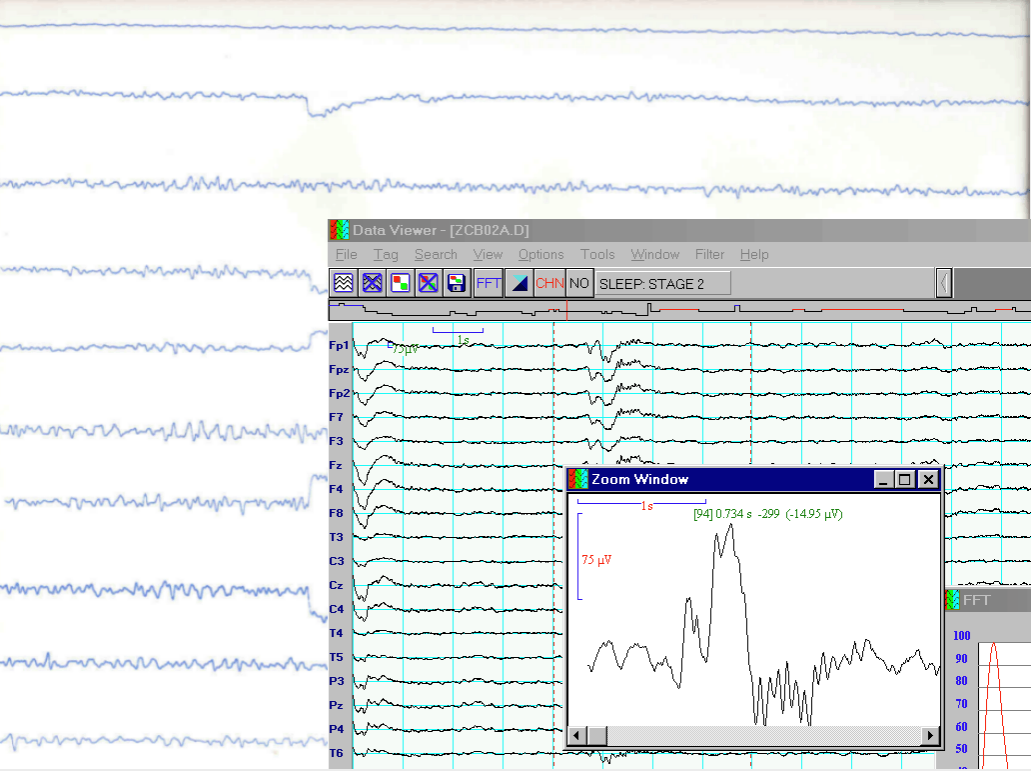
70 years of progress in clinical electroencephalography: from visual analysis of EEG traces on paper (background picture) to visual analysis of EEG traces displayed on CRT (front). Figure and caption reprinted from [12], © 2001 IEEE.

This situation, at least in relation to the clinical applications, was summarized in [12] as presented in Figure 1.

### 1.4 Univariate EEG analysis

This section briefly recalls the univariate linear and quadratic transforms, which were used in a significant amount of EEG studies.

### 1.5 Linear expansions

To explore the properties of an unknown signal *f *– in the absence of a quantitative model of its generation – we may use a linear expansion in a set of known functions {*g*_1_(*t*), *g*_2_(*t*),..., *g*_*N*_(*t*)}:



If {*g*_*i*_} form an orthonormal basis, *a*_*i *_are given simply by



Traditionally, orthogonal {*g*_*i*_} sets were limited to Dirac's deltas *δ*_*i *_and complex exponentials *e*^*iωt*^, corresponding to the time-domain and spectral analyses. In recent decades, new orthogonal bases were discovered, like e.g. wavelet packets or local cosines. The first and most popular orthogonal wavelet bases are built from dilations and translations of a single function . Only very special choices of *ψ *give an orthogonal basis, c.f. [13] – it was not before 1980. that first such orthogonal sets were discovered. Scaling a single function implies the characteristic "zooming property" of wavelets, that is increased time resolution and decreased frequency resolution at high frequencies. However, in the EEG analysis this seems to be more of a drawback than advantage.

To interpret (1), we usually relate those *g*_*i*_, that correspond to the largest |*a*_*i*_|, to some properties of *f*. For example, large value of  (spectral peak at *ω*_0_) suggests, that *f *contains periodic activity with base frequency near *ω*_0_. However, presence of the same periodic activity will not be so evident if |*a*_*i*_| are calculated e.g. in a wavelet basis, since in this case it will be diluted across several wavelets.

### 1.6 Time-frequency distributions

Increasing need to study the detailed picture of event-related changes of signal energy – e.g. in the event-related EEG (de)synchronization studies [14] or research on brain-computer interfaces [15] – turns our attention to the quadratic estimates of the time-frequency energy density. The most natural (at least for a physicist) is the Wigner-Ville transform :



However, it suffers from severe interferences, that is cross-terms. Cross-terms are the area of a time-frequency energy density estimate, which may be interpreted as indicating false presence of signal's activity in given time and frequency coordinates. Their origin can be deduced e.g. from equation (11). Several reduced interference distributions (RID) were proposed to minimize this drawback. It is achieved via the smoothing kernel *φ*, which decreases the influence of cross-terms at a cost of lower time-frequency resolution:



The most popular time-frequency distributions are spectrogram (squared modulus of the short-time Fourier transform) and scalogram (squared modulus of continuous wavelet transform). All of them can be derived from (4) with different kernels *φ*, and they give better or worse results for different types of signals. Therefore, it is possible to obtain a clear time-frequency picture of a given signal *f*, if we find an optimal kernel *φ *for its particular content. However, there is no general recipe for this choice. Also, these quadratic representations do not offer the direct parameterization of signal's features such as formula (1).

### 1.7 Adaptive time-frequency approximation

In the ideal situation we would like to have a linear expansion like (1), but with functions *g*_*i *_representing *all *the relevant structures present in *f*. Mere expansion of the set {*g*_*i*_} beyond a basis does not solve the problem. For the representation of each signal *f *we must *choose *the set functions {}*i *= 1..*M *adapted to represent its particular content. As a result, we get an approximation:



Approximation appears instead of the exact expansion, because to choose  from a set large enough to represent efficiently relevant signal's structures, we must give up the orthogonality. Adaptively chosen orthogonal representations, available in terms of wavelet packets, do not provide enough flexibility. Criterion of choice can be based upon the minimal norm of the residue. We can define an optimal *M*-approximation as an expansion, minimizing the following error *ε*:



Finding such an optimal approximation is NP-hard (intractable). It means that its computational complexity is growing with the size of the problem (in this case dimension of the signal) exponentially – in computer science this term is used as synonym of "faster than any polynomial". Such problems, even for moderately-sized input data, require times easily exceeding e.g. the age of the Universe.

We can formulate an almost equivalent decision problem, asking whether for given *D*, *ε*_1 _and *f *exists an *M*-approximation giving error (6) smaller than *ε*_1_. It can be proved to belong to the NP-complete class [16], that is problems possesing the two following properties [17]:

• Finding a solution requires exponential (non-polynomial time), but given solution can be checked in polynomial (P) time. The name "nondeterministic-polynomial" stems from the lack of a deterministic algorithm finding this answer in polynomial time – "guessing" the right answer is non-deterministic.

• Solution of each of the problem from the NP-class can be translated into solution of any other problem from this class in a polynomial time.

This class encompasses a variety of important problems, from the classical traveling salesman problem to e.g. factorization of large numbers, which has direct implications in cryptography. It is generally believed, that NP-complete problems do not have polynomial-time solutions, yet this basic theorem (*P *≠ *NP*) is still not proved (it may be also undecidable from the currently applied axioms).

Therefore, we rely on a suboptimal solution, which can be found by means of an iterative procedure like the matching pursuit algorithm (MP), proposed in [18]. However, even this sub-optimal solution is computationally intensive, so the first practical applications were not possible before the last decade.

### 1.8 Matching pursuit algorithm

In the first step of MP, the waveform  which best matches the signal *f *is chosen from the dictionary *D*. In each of the consecutive steps, the waveform  is matched to the signal *R*^*n *^*f*, which is the residual left after subtracting results of previous iterations:



Orthogonality of *R*^*n*+1 ^*f *and  in each step implies energy conservation



For a complete dictionary the procedure converges to *f*



### 1.9 Time-frequency dictionaries of Gabor functions

Time-frequency dictionaries are composed – apart from the complete Dirac and Fourier bases – from the Gabor functions, since these functions provide optimal joint time-frequency localization [13]. Real valued Gabor can be expressed as



where *K*(*γ*) is such that ||*g*_*γ*_|| = 1. Parameters *γ *= {*u*, *w*, *s*} of the possible Gabor functions constitute a 3-dimensional (phase *φ *is optimized separately in practical implementations), continuous space, from which a finite dictionary must be chosen for an implementation of the procedure (7). Originally (in [18]), dictionary's parameters were chosen from predefined dyadic sequences. However, any fixed scheme of subsampling the space of possible dictionary's functions leads to a statistical bias of the resulting decompositions. A solution proposed in [19] relies on stochastic dictionaries, in which parameters {*u*, *w*, *s*} are drawn from uniform distributions across ranges defined by sizes of the signal and the dictionary.

### 1.10 Estimate of signal's energy density

If we find the signal's approximation (5), we can also solve the problem of cross-terms present in the distributions from section 1.6. Namely, calculating the Wigner distribution of expansion (5) would yield



where  (, ) is a cross-Wigner transform of  and  given by



Double sum in (11) contains all the cross-terms. Owing to the representation (5), we can omit them explicitly and construct the time-frequency representation of signal's energy density from the first sum, containing auto-terms:



Energy conservation of this distribution is easily demonstrated (c.f. [18]).

## 2 Unification

In this section we collect and discuss the statements, needed for the acceptance of the thesis formulated in the Introduction:

Adaptive time-frequency approximations of signals unify most of the univariate computational approaches to EEG analysis, and offer compatibility with its traditional (visual) analysis, used in clinical applications.

### 2.1 *Lemma*: Matching pursuit sub-optimal solution to the problem of adaptive approximation is suitable for EEG analysis

**Figure 2:**
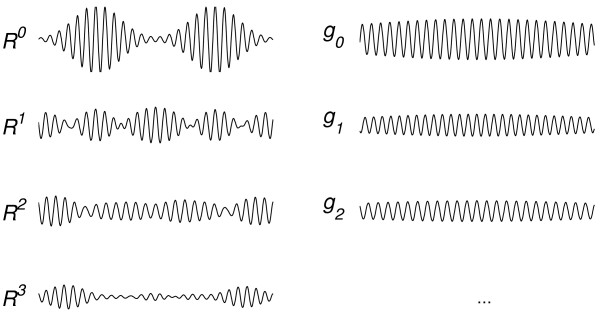
A failure in feature extraction: *R*^0 ^– analyzed signal, *g*_0 _– function fitted in the first iteration by the MP algorithm. Horizontal – time, vertical – amplitude, both in arbitrary units (simulated signals).

Sub-optimal solution of an intractable problem (section 1.7) must have its price. Mathematical examples of failures in pattern recognition, due to the greedy strategy (7) applied by the matching pursuit, were presented e.g. in [20,21]. These cases did not relate to structures encountered in biomedical signals, but the lack of a relevant counterexample does not deny it's existence. Figure 2 gives an example of a case referring directly to the transient oscillatory activity of the kind which may be actually present in the EEG [22].

Signal (*R*^0^) in Figure 2 is composed from two Gabor functions, both of them actually present in the dictionary *D *used for the decomposition (Section 1.9). In spite of that, we observe (in the right column) that the first function(*g*_0_) fitted to the signal is completely different from either of the two functions, from which the signal was composed! According to (7), MP algorithm has chosen function *g*_*i *_giving the largest product  in a *single *step. Of course, taking into account the next steps, this decision was definitely not optimal. Choosing the two Gabors, which were exactly represented in *D*, would explain 100% of signal's energy in only 2 iterations, contrary to the residues produced in the left column of Figure 2 as a consequence of the first choice.

However, such an effect occurs only if both Gabors present in the signal have not only the same frequencies, but also *exactly *the same phase. Such a "coincidence" would be possible in a biological signal *only *if both the structures were produced by the same generator. And still, MP represents them jointly only if their time centers are close enough. Their larger displacement would result in separate representation even in such a synchronized case. Therefore, we may argue that this effect, mathematically classified as a failure of the sub-optimal procedure, is actually a welcome feature in the analysis of physiological signals. It was explored in [23] for detecting the synchronized spiking activity, as opposed to series of unrelated EEG spikes.

Even if we were able to calculate the optimal adaptive decomposition minimizing error (6), it would have one more disadvantage: namely, the set of *M *functions  chosen to optimally represent signal *f*, may significantly differ from the optimal set of *M *+ 1 functions chosen for the same signal *f *(from the same dictionary *D*). With the iterative MP solution (7), {}*i *= 1..*M *will be the same in both decompositions. Considering that the features related to the sub-optimality of the MP solution turn to be advantages rather than drawbacks in the analysis of biomedical signals, we conclude that matching pursuit algorithm is the correct implementation for an adaptive time-frequency approximation of EEG.

### 2.2 *Lemma*: Matching pursuit decomposition is asymptotically well defined and does not depend on arbitrary settings

Calculating representation (5) of a signal *f *via matching pursuit in given dictionary *D *is a deterministic procedure, given by (7). Therefore, for a given signal, representation (5) depends only on the dictionary *D*. For the Gabor dictionaries discussed in section 1.9, the only free parameter is their size. It influences MP decomposition in a predictable way: larger dictionaries improve the quality of the decomposition, that is less waveforms are needed to achieve the same error *ε *in (6). However, starting from a certain density of dictionary's waveforms in the space of their parameters {*u*, *w*, *s*}, this improvement reaches asymptotically a saturation. At this point, which we shall tentatively term a "sufficient density of a Gabor dictionary", decompositions in larger dictionaries, or in different stochastic realizations of the same dictionary, are indistinguishable (of course this relates only to the structures coherent with the dictionary, c.f. [18]).

This qualitative reasoning – although still not backed up by a quantitative proof – is well supported by numerical experiments and years of real-world applications. Problems with mathematical derivations stem from the non-linearity of the MP procedure (7) and variable content of the analyzed signals. Therefore, the notion of "sufficient density" of a Gabor dictionary is up to now defined only empirically. Nevertheless, for the following we shall use it as a requirement for (5). For reasonable dimensions of signals, such densities are achieved with the dictionary sizes easily implementable on today's computers.

### 2.3 *Conjecture*: Relevant content of the EEG signal is coherent with Gabor time-frequency dictionaries

This statement depends on the meaning of "relevant EEG content", which, unfortunately, does not have a strict definition.

EEG originates from postsynaptic potentials of firing neurons; only if a large ensemble is working synchronously, the signal can reach a level measurable from the scalp electrodes. A single electrode records a potential from an arbitrary number of such ensembles, spatially filtered by different conductivities of cortex, dura mater, skull and scalp. Recorded EEG is not only affected by possibly non-linear interactions between these ensembles, but the signal may also contain extra-cerebral – biological or not – potentials (artifacts).

Rhythmic activity has been recognized as a prominent feature of the signal since the beginnings of EEG recordings. It appears as transient – rather than permanent – oscillations. These oscillations are produced by large masses of neurons (typically 10^4 ^– 10^7 ^[24]). Synchronization and desynchronization of these neural masses cannot be instantaneous, so the oscillations exhibit some waxing and waning. Gabor functions provide a concise general model for such signals.

"Relevant content of EEG" can be also translated to "relevant structures used to date in EEG analysis". That would refer mainly to the visual analysis, discussed in lemma 2.6 and 2.7.

Finally, non-permanence of this statement – underlined by using "conjecture" rather than "lemma" – relates also to the fact that, if any other structures would appear to be important in this context, they can be added to the dictionaries without a negative impact on the convergence of the MP procedure (7).

### 2.4 *Conjecture*: All the relevant EEG structures, which can be found via linear expansions in different bases, are included in the MP parameterization

As discussed in section 1.5, exploratory value of linear expansions relates to *g*_*i *_with the largest weights in expansion (1) – only those are usually treated as reflecting the significant signal's structures. Accepting Conjecture 2.3 we assume, that all relevant EEG structures are well represented in the Gabor dictionary. Also, waveforms used in linear expansions (1) can be more or less exactly assimilated by smooth functions (10). E.g. some wavelets contain discontinuities, but in the context of spatially-filtered EEG (c.f. Lemma 2.3) smooth Gabor functions should be more adequate for its description. Therefore, we may expect that if a signal contains a mixture of oscillatory and transients activities, and each of them would be highlighted in a different expansion (1), then all of them will be efficiently represented in the MP approximation (5).

As an example, we may quote a study of pharmaco-EEG [25], where traditional spectral estimators of spindling activity between 12 and 14 Hz were replaced by the energy of relevant structures estimated from MP expansion. It provided full compatibility with the traditional approach and better accordance with physiological expectations, owing to the increased sensitivity of MP-based estimator.

However, the correspondence to which we refer in this Conjecture, relates only to EEG *structures*, which should be parameterized by the MP decomposition as efficiently as in any of the linear expansions (1). But certain *statistical *properties of the signal can be derived only from its full representation e.g. in wavelet or Fourier bases. On the other hand, properties of MP expansion can be used to construct completely new measures, like e.g. the Gabor atom density (GAD), proposed for prediction of epileptic seizures in [26].

### 2.5 *Lemma*: Matching pursuit provides the most general solution to the problem of non-unique estimates of time-frequency energy density, applicable for EEG analysis

All the time-frequency distributions from section 1.6 are estimates of the same magnitude, that is energy density of the signal. However, estimates derived from different approaches (e.g. Wigner, RID, CWT or spectrogram) differ significantly, mainly due to the variable contribution of cross-terms. Also, estimates obtained from the same approach may look quite different depending on their parameters, like e.g. the length of the time window in spectrogram, different wavelets in CWT or smoothing kernels *φ *in RID.

Estimate (13), derived from the MP decomposition, contains the auto-terms, corresponding to structures present in expansion (5), and no cross-terms (section 1.10). Therefore, for a signal coherent with the dictionary used in the MP decomposition, estimate (13) approximates the intersection of its different quadratic estimates of energy density. This common part corresponds to the generalized auto-terms, contrary to the cross-terms variable between estimates. Therefore, if we accept Conjecture 2.3, this lemma is also proved. Even if we doubt the coherence of EEG signal with the Gabor dictionary, this approach is currently the best candidate for a unifying approach, owing to the uniqueness discussed in Lemma 2.2, since all the other quadratic estimates depend on an arbitrary choice of parameters.

Recent studies of brain electrical activity, recorded from the scalp [14,27] and from the cortex [28], confirmed also that (13) provides resolution superior to the previously applied quadratic methods.

### 2.6 *Lemma*: Among the contemporary methods of signal analysis, adaptive approximations with time-frequency dictionaries provide the best correspondence to visual EEG analysis

The knowledge, verified through decades of clinical applications, consists mainly of associations between appearances of rhythmic activities and certain waveforms in EEG with physiologically relevant states or pathologies, like e.g. level of alertness, sleep depth or epileptic seizure. These rhythms and waveforms constitute the dictionary of clinical EEG analysis. Due to a limited repeatability of their visual detection, the field is often considered an art rather than science [24]. Mapping this dictionary into mathematical terms will allow for application of repeatable scientific methods.

**EEG rhythms **(*δ*, *θ*, *α*, *β*, *γ*) are approximately assigned to frequency bands. Therefore, if we assume that those frequency bands are fixed, we can detect their presence from the relative power of the corresponding frequency band in the Fourier spectrum. However, power spectrum is a property of the whole analyzed epoch, and does not provide the information on the time extent of the given oscillatory phenomenon.

Appearance and disappearance of a rhythm can be clearly detected from a time-frequency map of signal's energy density, obtained from one of the quadratic methods discussed in section 1.6. However, stating that oscillations with frequency *f *last from the time *t*_1 _to *t*_2 _requires an analysis (post-processing) of a redundant map. Results will depend on the combination of the applied post-processing method and the chosen estimate of the energy density.

On the contrary, unequivocally defined (Lemma 2.2) MP expansion (5) gives *explicitly *the time span and frequency of detected oscillations.

**Transient EEG structures **– sleep spindles, K-complexes, epileptic spikes and many others – are obviously undetectable from the spectral estimates. They may exhibit certain, more or less typical, time-frequency signatures; we may imagine using the complex transforms (3) or (4) for their detection, but as previously mentioned we are confronted with continuum of possible transforms and post-processing algorithms.

Adaptive approximations (5) provide today the only method of an *explicit *description of a significant variety of waveforms. As presented in [23], this description is efficient also for non-oscillating structures like epileptic EEG spikes.

### 2.7 *Conjecture*: The correspondence from Lemma 2.6 can bridge the art of visual EEG analysis and reproducible methods of signal processing

Adaptive time-frequency approximation is currently the only signal processing method, offering description of structures, present in a signal, explicitly in terms of their physical parameters like amplitude, frequency or time width. Similarly to the way in which an expert evaluates EEG, the algorithm concentrates on most prominent, local structures, rather than general properties of the whole analyzed epoch.

Visual analysis of EEG relies primarily on detecting occurrences of rhythms and other waveforms, called graphoelements. In some cases, their classical definitions are given already in time-frequency terms. For example, sleep spindles are defined (c.f. [11]) as structures of least 0.5 sec duration, frequency between 12 and 14 Hz and amplitude above 15–25 *μV*. Such a definition can be directly applied to filter the decomposition (5) in the space of the parameters of functions , fitted to the analyzed signal. Waveforms , conforming to such ranges of parameters, represent sleep spindles – as verified by comparison with their visual detection in [29].

However, not all the graphoelements have such strict, mathematical definitions. The very name "graphoelement" (rather than waveforms) suggests, that their visual detection may rely on different aspects of its shape. Nevertheless, most of these shapes can be effectively approximated by Gabor functions. In such cases we may a posteriori adapt the ranges of the parameters of , to maximize the concordance with visual detection. This resembles the classical paradigm of expert systems, however, using the MP parameterization (5) has the major advantage of operating in the space of physically meaningful and universal parameters, like time width, amplitude or frequency. An example of a successful implementation of such an approach and its broader discussion can be found in [23].

## 3 Discussion

Sections 2.3, 2.4 and 2.7, necessary for the strict proof of the main thesis, are currently conjectures rather than lemmas. Before we discuss this issue any further, let us gain a proper perspective by considering another theorem, which is generally *believed *to be true, and, although still not proved, is used by most of us on a daily basis:

As mentioned in section 1.7, we still do not have a strict mathematical proof, that the problems belonging to the *NP *class do *not *have a polynomial-time solution (*P *≠ *NP*?). Factorization of large numbers also belongs to the *NP *class. Strength of the public key cryptography depends on factorization. Therefore, if for any problem from the *NP *class, a polynomial-time solution is found – which is, in theory, not impossible – it can be used to break the contemporary public key cryptography. Nevertheless, we are still using this system for securing transmission of sensitive data (e.g. credit card numbers) over Internet. Our belief is based upon the lack of counterexamples, experimental evidence given by years of application, and up-to-date security offered by these methods.

## 4 Conclusion

Similarly to the above example, the main thesis of this Dissertation is not yet backed up by a rigorous proof, because:

• Conjecture from Section 2.7, stating that proposed approach *can bridge the art of visual EEG analysis and reproducible methods of signal processing*, needs to be confirmed on a larger number of cases, and by independent opinions of the masters of the art of electroencephalography.

• A complete model, which would explain in details the generation of all the relevant aspects of the EEG time series, still does not exist. Without a strict definition of the word *relevant *in this context, we cannot construct a rigorous proof of conjectures from Sections 2.3 and 2.4, which relate to the relevant content of the EEG time series.

Up to now, all the experiments and simulations confirm the main thesis. Its acceptance and wide implementation will bring a direct application of the indispensable knowledge gathered in decades of visual analysis and a significant improvement of the efficiency of EEG-related research. Hopefully, this may contribute to the renaissance of electroencephalography in research and clinical applications.
